# The impact of retirement on blood pressure: evidence from a nationwide survey in China

**DOI:** 10.1186/s12889-024-18422-z

**Published:** 2024-06-11

**Authors:** Jiarun Mi, Xueyan Han, Man Cao, Hanchao Cheng, Zhaoyang Pan, Jian Guo, Wei Sun, Yuanli Liu, Congyi Zheng, Xin Wang, Xue Cao, Zhen Hu, Yixin Tian, Zengwu Wang, Tianjia Guan

**Affiliations:** 1 School of Health Policy and Management, Chinese Academy of Medical Sciences and Peking Union Medical College, Dongdansantiao, Dongcheng district, Beijing, 100730 China; 2 Division of Prevention and Community Health, National Center for Cardiovascular Disease, National Clinical Research center of Cardiovascular, Disease, State Key Laboratory of Cardiovascular Disease, Fuwai Hospital, Peking Union Medical College & Chinese Academy of Medical Sciences, Beijing, China; 3 Department of Cardiology, State Key Laboratory of Complex Severe and Rare Diseases, Peking Union Medical College Hospital, Chinese Academy of Medical Sciences & Peking Union Medical College, Beijing, 100730 China; 4 Medical Research Center, State Key Laboratory of Complex Severe and Rare Diseases, Peking Union Medical College Hospital, Chinese Academy of Medical Sciences & Peking Union Medical College, Beijing, 100730 China

**Keywords:** Retirement, Health impact, Blood pressure, Regression discontinuity design

## Abstract

**Introduction:**

The health impact of retirement is controversial. Most previous studies have been based on self-reported health indicators or the endpoints of some chronic diseases (e.g., morbidity or mortality), but objective physiological indicators (e.g., blood pressure) have rarely been used. The objective of this study is to elucidate the health effects of retirement on blood pressure, thereby offering empirical evidence to facilitate the health of retirees and to optimize retirement policies.

**Methods:**

From 2012 to 2015, 84,696 participants of the Chinese Hypertension Survey (CHS) were included in this study. We applied the fuzzy regression discontinuity design (FRDD) to identify retirement’s causal effect on systolic blood pressure (SBP), diastolic blood pressure (DBP) and pulse pressure. We also explored the heterogeneity in the effects of retirement across different sex and education level groups.

**Results:**

Based on the fully adjusted model, we estimated that retirement increased SBP by 5.047 mm Hg (95% CI: -2.628-12.723, *P* value: 0.197), DBP by 0.614 mm Hg (95% CI: -3.879-5.108, *P* value: 0.789) and pulse pressure by 4.433 mm Hg (95% CI: -0.985-9.851, *P* value: 0.109). We found that retirement led to a significant increase in male participants’ SBP and pulse pressure as well as a possible decrease in female participants’ blood pressure. Additionally, the blood pressure levels of low-educated participants were more vulnerable to the shock of retirement.

**Conclusion:**

Retirement is associated with an increase in blood pressure level. There is a causal relationship between the increase in blood pressure levels of men and retirement. Policy-makers should pay extra attention to the health status of men and less educated people when adjusting retirement policies in the future.

**Supplementary Information:**

The online version contains supplementary material available at 10.1186/s12889-024-18422-z.

## What is already known on this topic?

Retirement has been considered as a crucial transition in life and may affect health through several different pathways.

## What this study adds?

On average, retirement was associated with a possible increased blood pressure in China. There was gender and educational level heterogeneity in the effect of retirement on blood pressure. Retirement led to a significant increase in male participants’ blood pressure level but a possible decrease in female participants’ blood pressure level.

## How this study might affect research, practice or policy?

When adjusting retirement policies (e.g., delaying retirement), policy-makers in China should pay extra attention to the health status of men and less educated people, because their blood pressure level were more vulnerable to retirement’s impact.

## Introduction

Retirement is generally defined as withdrawing from a job or occupation and no longer actively seeking employment, i.e., permanently leaving the labor market. As a crucial transition in life, retirement may affect health through several pathways, such as changing health behaviors [1, 2] and mental stress [3]. With increasing life expectancy and an aging population, clarifying the association between retirement and health could be helpful to optimize retirement policies and medical resources at the national level.

Despite the growing interest in research on the health effects of retirement, the conclusions of existing studies remain inconsistent, variously reporting beneficial [4, 5], harmful [6–8] and insignificant effects [3]. For instance, Coe and Zamarro (2011) used the Survey of Health, Ageing and Retirement in Europe (SHARE) data and found that retirement led to a 35% reduction in the probability of self-reported poor health, affirming the positive health effects of retirement [9]. Similarly, other researchers also used SHARE to further support that retirement had a positive impact on mental health and subjective health [10, 11]. In contrast, several studies applied a longitudinal design and found that retirement, especially involuntary retirement, could have an adverse effect on physical and mental health [6, 8]. Some studies failed to find any significant health effect of retirement, especially when accounting for the endogeneity of retirement decisions [12, 13].

Additionally, previous studies regarding the health impact of retirement focused on different health outcomes, most of which were based on self-reported health indicators or the endpoints of some chronic diseases (e.g., morbidity or mortality) [5], but objective physiological indicators (e.g., blood pressure) were rarely used. Selecting such indicators as the outcome could avoid the bias associated with participants’ subjective reporting in the survey. Among these indicators, blood pressure is an excellent indicator of overall health, and elevated blood pressure is a significant modifiable factor for many chronic diseases, especially cardiovascular diseases. Therefore, studying the impact of retirement on blood pressure is helpful to design prevention strategies against adverse cardiovascular outcomes for retirees. When exploring the health effects of retirement on blood pressure or hypertension, considering reverse causality is necessary because poor cardiovascular status may be a determining factor in retirement or even early retirement [14].

Therefore, in this study, we applied a quasi-experimental approach, the regression discontinuity design (RDD), aiming to explore the causal effect of retirement on systolic blood pressure (SBP), diastolic blood pressure (DBP) and pulse pressure. In the observational study, RDD is an approximation to a randomized controlled trial, which allows the causal effect to be estimated in the absence of an explicit randomization treatment and could effectively avoid the reverse causality problems. We included participants from a nationally representative population study conducted in China.

## Methods

### Study design and population

The population in our study was derived from the Chinese Hypertension Survey (CHS), which covered 262 counties from all 31 provinces of mainland China from 2012 to 2015. Briefly, the CHS applied a stratified multistage random sampling method to obtain a nationwide representative sample of the Chinese general population aged ≥ 15 years. More details about the design, method and participants of the CHS have been elaborated elsewhere [15, 16].

### Retirement definition and sample selection criterion

The statutory retirement policy in China was officially established in 1978 and has not been changed since then [3]. Currently, the age of statutory retirement in China is 60 years for men, 55 years for female civil servants and 50 years for other female employees. This age does not vary by province and is mandatory in public sectors but not mandatory in private sectors. Since China’s statutory retirement age is also pension eligibility age, employees of private sectors might be motivated to retire at the statutory retirement age because they become eligible for a pension [17].

Two questions in the CHS questionnaire were used to determine retirement: (1) employment status (1=employed, 2=retired, 3=school student, 4=unemployed) and (2) occupation (retirees fill in the occupation before retirement). The specific response options of question (2) are: 1=Administrators in government or institutions; 2=Professional and technical personnel; 3=Clerical staff; 4=Service industry workers; 5=Individual business operators; 6=Non-agricultural industrial workers; 7=Farmers engaged in non-agricultural labor; 8=Agricultural workers (involved in farming, forestry, animal husbandry, and fishery); 9=Others. In China, individuals engaged in occupations 4-9 are considered to be working in the private sector, where the implementation of statutory retirement policies is not strict, or the concept of retirement may even be absent. Therefore, they were excluded from our study. Participants were defined as retired if they self-reported that they have retired (question (1)).

### Outcomes and covariates

We used systolic blood pressure, diastolic blood pressure and pulse pressure as the health outcomes of this study. After the participants rested for at least 5 minutes, the blood pressure was measured three times on the right arm positioned at heart level by a well-trained physician with the help of an OMRON HBP-1300 Professional Portable Blood Pressure Monitor. Additionally, the time interval between each measurement was at least 30 s, and we used the average of three readings for our analysis. The pulse pressure is obtained by calculating the difference between systolic and diastolic blood pressure.

We collected the covariates of participants using a face-to-face standardized questionnaire of the CHS. The covariates included (1) sociodemographic variables: sex, age, education level and marriage; (2) health behavior variables: smoking and alcohol consumption; and (3) taking antihypertensive drugs. Participants with a senior high school diploma or higher were defined as highly educated.

### Statistical analysis

We applied a regression discontinuity design (RDD) to estimate the association between retirement and health outcomes, which can be used when a continuous assignment variable features a threshold to determine whether an individual receives a treatment or not [18]. Specifically, RDD is suited for scenarios that evaluate the effect of an external intervention based on a clear threshold of a continuous variable (i.e., the assignment variable). When subjects are naturally divided into two groups—those receiving the intervention and those not—based on this explicit threshold of the continuous variable, RDD leverages information around the threshold to estimate the intervention's effect through a local regression. This design is particularly apt for assessing the impact of policy interventions, legal enactments, or educational programs. The key advantage of RDD lies in its ability to provide causal inferences closer to those of randomized experiments than observational studies can. This is because it assumes that individuals just below and just above the threshold are similar in all respects except for their intervention status, thereby minimizing the influence of potential confounders. This makes RDD a powerful tool for researching the effectiveness of policies or intervention measures.

In this study, participants’ age is such a continuous assignment variable and can decide the external treatment status (i.e., retirement). RDD is categorized into two types: Sharp RDD (SRDD) and Fuzzy RDD FRDD). From a probabilistic perspective, in Sharp RDD, the probability of receiving treatment jumps from 0 to 1 at the threshold, meaning assignment to treatment or control is deterministic based on the threshold. Fuzzy RDD posits that the likelihood of individuals receiving an intervention upon crossing a predefined threshold does not shift from 0 to 1 but rather experiences a probabilistic increase. This indicates that not all individuals surpassing the threshold will necessarily receive the intervention, as treatment assignment is probabilistic rather than absolute. Such a design mirrors real-world scenarios where individuals may respond differently to potential interventions or policies, rendering RDD analysis more attuned to practical situations. Given that the change in the probability of retirement at the statutory retirement age is not from 0 to 1, FRDD was adopted in this study.

Under the assumptions of RDD, individuals just below and just above the threshold are similar in both observed and unobserved characteristics, making RDD near the threshold akin to a randomized controlled trial. This key characteristic of RDD, in addressing the issue of omitted variable bias, reduces the need to directly control for potential confounding factors, as long as the design's assumptions hold. Although we cannot observe the distribution of all characteristics on both sides of the threshold, typically, two tests can be conducted to ensure the validity of RDD. The first test is that the assignment variable has not been manipulated (i.e., CHS participants did not report a false age to change their retirement eligibility), which ensures that the allocation of the treatment status to participants near the threshold of the assignment variable is an entirely random process [19]. The second test is that there is no discontinuous change in the covariates at the threshold of the assignment variable, which could have an effect on the outcomes and be incorrectly attributed to the intervention [18]. It ensures that individuals near the threshold on both sides have similar observable characteristics. For the first test, we conducted a formal statistical test of the manipulation in assignment developed by McCrary in 2008 [20]. For the second test, referring to previous studies [21, 22], we used covarites as outcome variables of RDD to test their continuity at the threshold. These two assumptions ensure the exchangeability of participants just above and just below the threshold. In other words, these two groups are homogeneous. We present the results of these two tests in the "the validity of the FRDD" section.

Since RDD relies on samples within a range around the threshold to estimate the treatment effect, it depends on the determination of this range, also known as the bandwidth. Bandwidth is a critical parameter in RDD, defining the data range around the threshold. This range is used to estimate the local effect of the intervention. Choosing the appropriate bandwidth is crucial, as it determines the sensitivity and precision of the analysis. A bandwidth too large may include information irrelevant to the intervention and introduce noise, thereby violating the assumption of interchangeability of samples near both sides of the threshold. Conversely, a bandwidth too small may lead to the omission of important local information, reducing the statistical power of RDD. With the assistance of the R package rdrobust, we adopted the bandwidth algorithm developed by Imbens and Kalyanaraman [23], recommended in previous RDD research and considered the optimal method for calculating bandwidth. According to this calculation, the optimal bandwidth for this study's RDD was set at 48 months. In the robustness checks, we also used 50%, 80%, 120% and 150% of the bandwidth to check whether our results were sensitive to bandwidth selection.

Considering that female civil servants are a relatively small group [24], we used age 50 as the threshold of RDD for all women according to previous studies [3]. Because of the difference in the statutory retirement age between men and women, we standardized for age to explore the health effect among the full population in the FRDD. We defined the standardized age (SA) as the actual age in months minus the corresponding statutory retirement age for each sex: SA = age in months - 720 for men, and SA = age in months - 600 for women. SA was the assignment variable for the FRDD in this analysis, and its threshold was 0.

The FRDD applied a two-stage regression equation, and its model was set as follows:

first-stage regression equation:

$${Retirement}_{i}={\alpha }_{0}+{\alpha }_{1}{Above}_{i}+{\alpha }_{2}\psi ({SA}_{i})+{\alpha }_{3}{Above}_{i}\psi ({SA}_{i})+\gamma {X}_{i}+{\mu }_{i}$$

second-stage regression equation:

$${Y}_{i}={\beta }_{0}+{\beta }_{1}\widehat{{Retirement}_{i}}+{\beta }_{2}\psi ({SA}_{i})+{\beta }_{3}{Above}_{i}\psi ({SA}_{i})+k{X}_{i}+{\varepsilon }_{i}$$

The first-stage regression equation is used to estimate the impact of crossing the statutory retirement age threshold on retirement. Through this regression, we calculate whether there is a statistically significant discontinuous change in the retirement rate upon crossing the threshold, as well as the magnitude of this change. Since the second-stage regression is based on the results of the first stage, it is crucial to ensure a significant discontinuous change in the retirement rate in the first-stage regression. Consequently, we present the results of the first-stage regression in the “discontinuous change in retirement rate” section. ${Retirement}_{i}$ denotes whether participant $i$ is retired (according to their response in the CHS), and ${Y}_{i}$ is the health outcome of this study. ${Above}_{i}$ is a dummy variable and is equal to 1 if participant $i$ is older than the statutory retirement age. $\psi ({SA}_{i})$ is a polynomial for SA. $\widehat{{Retirement}_{i}}$ is the fitted value from the first-stage regression equation. ${X}_{i}$ is a vector of covarites of this study. The perturbation terms are represented by ${\mu }_{i}$ and ${\varepsilon }_{i}$. The estimation of ${\beta }_{1}$ is the health impact of retirement.

Following the advice of econometricians, we used nonparametric local linear regression in the main analysis to estimate the health effect of retirement, which could reduce the risk of data overfitting compared to higher-order polynomials [22, 25]. In the robustness checks, we also added quadratic terms to the FRDD model. In all analyses, we applied a triangular kernel function to give more weight to samples closer to the threshold. The statistical analysis was performed using R software (version 4.2.0), and a two-tailed *p* value of < 0.05 was considered to be statistically significant.

## Results

### Sample characteristics

The sample characteristics of 84,696 participants included in our study are shown in Table 1. The median age of all participants was 40.58 years, with an interquartile range of 30.50-55.08. Within the optimal bandwidth of 48 months, there were 8,127 participants with a median age of 53.50 years (interquartile range: 49.75-59.75). Among participants within the optimal bandwidth, 39.5% had retired, 96.3% were married, 58.6% had received at least a senior high school education, 42.5% had normal BMI and women accounted for 52.4%. The median SBP was 127.33 mm Hg (interquartile range: 117.33-139.33), and the median DBP was 78.00 mm Hg (interquartile range: 71.33-84.33).

**Table 1:** Sample characteristics of 84,696 participants included in our study

Variables	Full sample **(*N*=84,696)**	Sample within optimal bandwidth **(*N*=8,127)**	Sample below threshold within optimal bandwidth **(*N*=4,079)**	Sample above threshold within optimal bandwidth **(*N*=4,048)**
**Age in years, median (IQR)**	40.58 (30.50,55.08)	53.50 (49.75,59.75)	49.83 (47.83,57.83)	53.67 (51.67,61.75)
**Sex**				
Male	46727 (55.2%)	3868 (47.6%)	1947 (47.7%)	1921 (47.5%)
Female	37969 (44.8%)	4259 (52.4%)	2132 (52.3%)	2127 (52.5%)
**Retirement**				
No	65178 (77.0%)	4915 (60.5%)	3291 (80.7%)	1624 (40.1%)
Yes	19518 (23.0%)	3212 (39.5%)	788 (19.3%)	2424 (59.9%)
**Married**				
No	14080 (16.6%)	301 (3.7%)	138 (3.4%)	163 (4.0%)
Yes	70616 (83.4%)	7826 (96.3%)	3941 (96.6%)	3885 (96.0%)
**High-educated**				
No	26792 (31.6%)	3365 (41.4%)	1551 (38.0%)	1814 (44.8%)
Yes	57904 (68.4%)	4762 (58.6%)	2528 (62.0%)	2234 (55.2%)
**Smoke**				
No	65027 (76.8%)	6278 (77.2%)	3108 (76.2%)	3170 (78.3%)
Yes	19669 (23.2%)	1849 (22.8%)	971 (23.8%)	878 (21.7%)
**Drink**				
No	70289 (83.0%)	6631 (81.6%)	3325 (81.5%)	3306 (81.7%)
Yes	14407 (17.0%)	1496 (18.4%)	754 (18.5%)	742 (18.3%)
**Antihypertensive drugs**				
No	74911 (88.4%)	6625 (81.5%)	3433 (84.2%)	3192 (78.9%)
Yes	9785 (11.6%)	1502 (18.5%)	646 (15.8%)	856 (21.1%)
**BMI**				
Underweight	3656 (4.3%)	133 (1.6%)	72 (1.8%)	61 (1.5%)
Normal	41340 (48.8%)	3456 (42.5%)	1816 (44.5%)	1640 (40.5%)
Overweight	29026 (34.3%)	3401 (41.8%)	1657 (40.6%)	1744 (43.1%)
Obese	10674 (12.6%)	1137 (14.0%)	534 (13.1%)	603 (14.9%)
**Systolic blood pressure, median (IQR)**	123.00 (114.00,133.67)	127.33 (117.33,139.33)	126.33 (116.67,138.00)	128.33 (118.64,140.58)
**Diastolic blood pressure, median (IQR)**	75 (69.00,81.33)	78.00 (71.33,84.33)	77.67 (71.33,84.33)	78.00 (71.33,84.67)
**Pulse pressure, median (IQR)**	47.67 (41.00,55.48)	49.67 (42.67,57.67)	48.67 (42.33,56.67)	50.33 (43.33,59.00)

### Discontinuous change in retirement rate

**Fig. 1:**
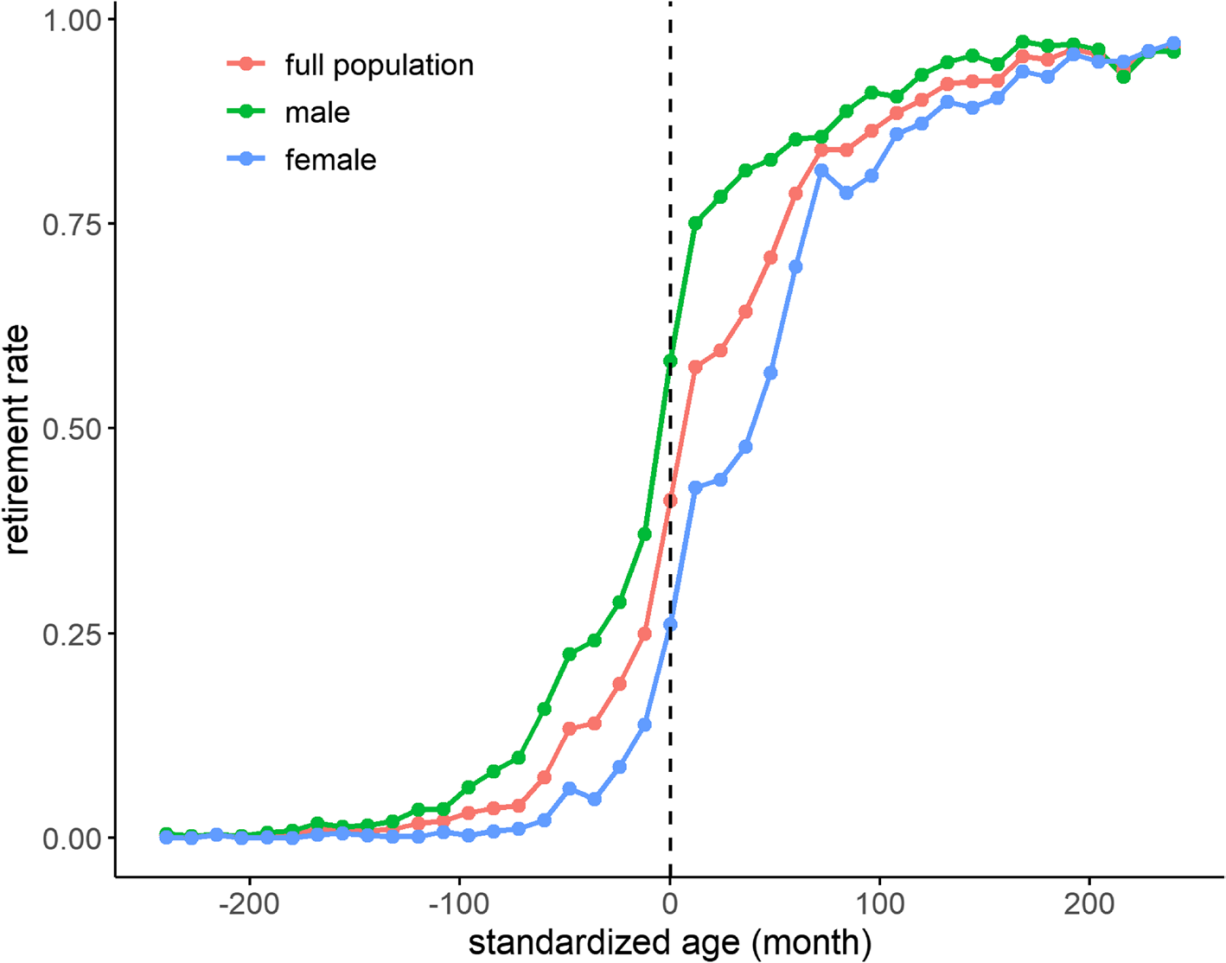
Retirement rate by standardized age

The changes in retirement rate by SA in the full population, men and women are shown in Fig. 1. At the threshold of SA, we found visual evidence of discontinuous changes in retirement rate among all three groups, indicating that the statutory retirement age was well enforced among participants included in this study. Specifically, there was a more significant jump at the threshold among male participants because their statutory retirement age was less complicated than that of female participants. We also presented the first-stage regression results of the FRDD in Table S1, which was formal evidence on the discontinuity of the retirement rate at the threshold. The statutory retirement policy significantly increased the retirement rate by 21.7% (95% CI, 0.174-0.262) for all participants, 22.6% for male participants (95% CI, 0.163-0.292) and 21.3% (95% CI, 0.159-0.267) for female participants.

### The validity of the FRDD

The results of the McCrary test for data manipulation are shown in Table S2. We failed to reject the null hypothesis that the density was smooth across the threshold, providing evidence of no manipulation of the assignment variable around the threshold. We used the covariates as outcome variables for the FRDD to check their continuity at the threshold, and the results are presented in Table S3. We did not find any significant discontinuous change at the threshold in all covariates.

### Impact of retirement in the full population

**Fig. 2:**
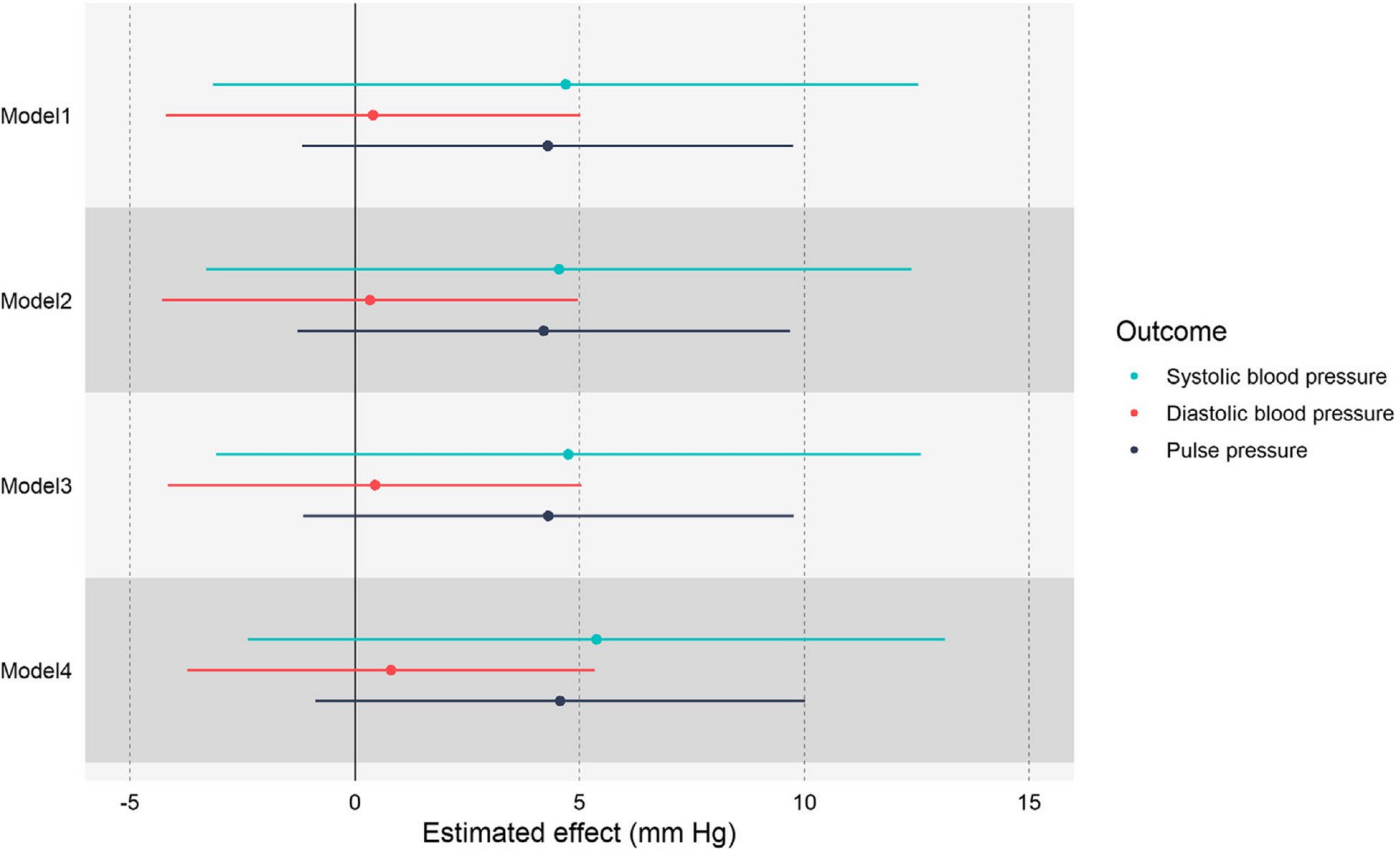
The FRDD estimates of the effect of retirement on health outcomes. Model 1 did not include any covariates. Model 2 adjusted for sociodemographic variables (i.e., education level and marriage status). Model 3 additionally adjusted for health behavior variables (i.e., smoking and alcohol consumption). Model 4 (i.e., full adjusted model) additionally adjusted for antihypertensive drug variables

Although retirement had an elevating impact on blood pressure based on FRDD estimates, this impact was not statistically significant. As shown in Fig. 2, based on the results of the full adjusted model (i.e., Model 4), we estimated that retirement increased SBP by 5.376 mm Hg (95% CI: -2.383-13.134, *P* value: 0.174), DBP by 0.805 mm Hg (95% CI: -3.731-5.341, *P* value: 0.728) and pulse pressure by 4.570 mm Hg (95% CI: -0.878-10.019, *P* value: 0.100). From Model 1 to Model 4, there was almost no difference in the estimated effect and its statistical significance, so the robustness of the results was also verified. Therefore, we failed to reject the null hypothesis that retirement had no effect on blood pressure in the full population on average.

### Heterogeneity of retirement effects across subgroups

We further explored whether the effect of retirement on blood pressure differed among some subgroups, and the results are shown in Table S4. We found that retirement had an opposite effect on blood pressure in men and women. Retirement may increase men’s blood pressure as well as reduce women’s blood pressure. Specifically, taking DBP as an example, the estimate of retirement’s effect was 4.334 mm Hg (95% CI: -2.022-10.691, *P* value: 0.181) in men and -2.896 mm Hg (95% CI: -9.358-3.566, *P* value: 0.380) in women. Additionally, compared to low-educated participants, we found that the effect of retirement on blood pressure in high-educated participants was close to the null value, and the *P* value was close to 1. We also observed that, although the outcomes for both groups were statistically insignificant, individuals on antihypertensive medication were more likely to experience adverse effects on blood pressure due to retirement compared to those not taking such medication. For DBP, retirement was associated with an increase of 3.583 mm Hg in individuals on antihypertensive medication (95% CI: -3.089, 10.255, *P* value: 0.293), whereas in individuals not on medication, the effect was only 0.340 mm Hg (95% CI: -4.555, 5.235, *P* value: 0.892).

### Robustness checks

The results of robustness checks are shown in Figure S1 and Table S5. Although the estimates of the effects decreased with increasing bandwidth, on average, they were close to our baseline estimates, and the statistical significance of effect estimates did not vary by bandwidth selection, which indicated the insensitivity to bandwidth of our results. Additionally, the similar results of using quadratic terms for age and bias-corrected estimates with robust standard errors of the FRDD further supported the robustness of our findings.

## Discussion

On average, we failed to find any significant impact of retirement on SBP, DBP and pulse pressure in the full participants. However, in the subgroup analysis, we found intergroup heterogeneity in the health impact of retirement. Specifically, retirement resulted in a significant increase in SBP and pulse pressure in men but a possible decrease in blood pressure in women. Compared to highly educated groups, the blood pressure of people with lower education was more vulnerable to retirement. The robustness of all results was verified by including different covariates, using different functional forms and changing the size of the bandwidth.

This work contributes to the causal evidence regarding the health impact of retirement on blood pressure in China. With the dramatic increase in life expectancy, China is confronted with the most rapid population aging all over the world [26], which has resulted in a looming crisis of the shrinking workforce and pension unsustainability. However, China’s current retirement age is one of the earliest in the world [3]. In response, policy-makers in China are planning to implement a series of reforms to delay retirement to break away from the formidable dilemma of population aging, as some developed countries have done before [27]. When adjusting for the current statutory retirement age, the health effect of retirement, especially the causal effect, should be a crucial consideration. In terms of health outcomes, hypertension remains a major health risk factor for retirees in China, where more than a quarter of older adults suffer from hypertension, and the prevalence has continued to increase during recent decades [28], resulting in a significant socioeconomic and health burden. Therefore, the causal effect of retirement on blood pressure should be a focus of policy-makers in China. Our research fills a gap in such causal evidence that has rarely been mentioned in previous studies in China.

### Insignificant increase in blood pressure among the full population

In all main analyses, retirement led to an insignificant increase in SBP, DBP and pulse pressure among the full population. As the size of the bandwidth increased, the impact on blood pressure-related outcomes decreased, which was consistent with a previous study on the impact of screening for hypertension [18]. In contrast to our findings, the few previous studies in China mostly confirmed the positive effects of retirement on blood pressure. For instance, a longitudinal study suggested that retirement was associated with a lower diastolic blood pressure and a slowdown in the increase in systolic blood pressure, and this association was strengthened among men and urban dwellers [29]. Similarly, another Chinese study also found that retirement predicted a decrease in SBP and DBP and a lower prevalence of hypertension (OR: 0.979, 95% CI: 0.968-0.990) [30]. Strategies for participant selection and the method for controlling retirement’s endogeneity could account for the difference in results across studies. Compared to other studies, we fully excluded participants in the private sector to ensure the validity of the statutory retirement threshold, and we used RDD to overcome endogeneity in retirement decisions to the maximum extent possible.

### Retirement effects across subgroups

Our results suggested that the impact of retirement on blood pressure varied by sex and education level. Retirement resulted in a significant increase in male SBP and pulse but was associated with a possible reduction in female blood pressure. Previous studies have shown that, compared to DBP, SBP is more closely associated with adverse cardiovascular outcomes and receives greater emphasis in Chinese Guidelines for the Management of Hypertension [18, 31]. Additionally, wide pulse pressure, as a potentially independent risk factor for many cardiovascular diseases, is not sensitive to traditional antihypertensive therapy targeting systolic and diastolic blood pressure [32]. Therefore, the significant causal impact of retirement on the increase in SBP and pulse pressure in men should be taken into account when implementing an adjustment for the statutory retirement age.

In contrast, male and less educated people’s blood pressure levels are more susceptible to the negative effects of retirement. Several possible mechanisms could account for our results. First, due to the transitions of social status, income levels and social activities, men might be more prone to mental illness after retirement, which has been identified as a potential risk factor for hypertension in some epidemiology studies [33, 34]. A recent study suggested that the probability of feeling lonely increased significantly among men after retirement but was insignificant among women [35]. Similarly, a longitudinal study in China also showed that retirement increased the risk of depression for retirees (OR: 1.5, 95% CI: 1.14-1.97) [36]. Second, because of the loss of work-related restrictions on lifestyle, male retirees are more likely to develop unhealthy habits, especially unhealthy eating habits. A previous study confirmed the sex-specific causal effect of retirement on eating behavior in that retirement led to reduced healthy eating (i.e., the consumption of fruit and vegetables) and an increased risk of obesity in men but had no effect in women [37].

For women, our study tends to believe that retirement may have a positive impact on their blood pressure. A previous study suggests that female employees, due to the dual factors of exposure to work stress and family caregiving responsibilities before retirement, often experience an increase in dynamic blood pressure, which may persist for several years [38]. Therefore, retirement could liberate women from the heavy work stress, potentially benefiting their blood pressure levels. However, some studies, based on other health outcomes (such as increased BMI [39], obesity [19], elevated blood lipids [40]), argue that retirement is detrimental to women's health, as these outcomes are potential risk factors for hypertension. Previous research indicates that marital status, number of children, and family caregiving significantly affect women's blood pressure levels. A study in Korea showed that married women with low income have the strongest correlation with hypertension (OR: 1.83, 95% CI:1.71, 1.97) [41]. Two other studies also noted that a larger family size (e.g., number of children) and caregiving responsibilities are often associated with increased variability in women's blood pressure or a higher risk of other metabolic syndromes [42, 43]. After retirement, women's labor burden related to family obligations may increase, which could be a potential reason why retirement might harm women's health. Medical professionals and policymakers should pay attention to the changes in women's health status after retirement, especially those who are married or have significant family caregiving responsibilities. Promoting health knowledge among women and providing mental health guidance to alleviate changes in women's post-retirement life and psychological stress conditions is a good practice. Additionally, men should take on more household chores after their spouses retire, which can help relieve the pressure of increased family caregiving responsibilities for women after retirement and promote a healthy transition to retirement. On the other hand, studies suggest that regularly undertaking household chores is also a good lifestyle for maintaining normal blood pressure levels for men [44]. For women themselves, maintaining a healthy lifestyle after retirement, especially in terms of dietary habits, is crucial. Some studies believe that compared to men, women prefer sweets more [45], so they might have more time to consume sweets after retirement, leading to increased blood sugar levels and thereby increasing the risk of chronic diseases [40].

Additionally, the differences in the size of retirement impact across different education level subgroups were consistent with our expectations. Due to the lack of health knowledge, less educated people have more difficulty maintaining a healthy lifestyle in the transition of retirement, giving rise to obesity or other risk factors for hypertension [1, 46].

Our study reveals that retirement has a substantially more adverse effect on blood pressure in individuals on antihypertensive medication than those not on such treatment. This observation can be rationalized through several mechanisms: Firstly, individuals on antihypertensive medication are indicative of pre-existing elevated blood pressure levels prior to retirement. Consequently, any fluctuations in external factors, such as economic and lifestyle changes triggered by retirement, when interacted with their ongoing medication regimen, could likely complicate the regulation of blood pressure, thereby exerting a more pronounced effect. Secondly, evidence from prior research suggests a correlation between retirement and a decline in medication adherence for chronic conditions, notably hypertension and diabetes [47, 48]. For instance, a Finnish study highlighted an increased risk of deteriorating adherence to antihypertensive medication post-retirement in both men (OR: 1.32, 95% CI: 1.03, 1.68) and women (OR: 1.25, 95% CI: 1.07, 1.46) [48]. Thirdly, from a psychological standpoint, individuals on antihypertensive drugs might be inherently more focused on their health due to their compromised health status, rendering them more susceptible to the stressors associated with life changes post-retirement. This heightened sensitivity could amplify the psychological stress's impact on blood pressure. These findings underscore the necessity for public health professionals and policymakers to closely monitor and address the blood pressure changes in individuals with pre-retirement hypertension. Enhancing health education and screening for blood pressure in this demographic is crucial to ensuring sustained medication adherence post-retirement.

### Public health relevence and policy implications

This study holds significant public health and clinical relevance. As China faces intensified aging of its population, nearly a quarter of the Chinese populace has entered retirement. Transitioning healthily into retirement is a pivotal aspect of life that impacts the well-being and tranquility of retirees' latter years. Therefore, examining the health impacts of retirement is crucial as it aids in optimizing the allocation of medical resources to facilitate a healthy retirement transition for diverse subpopulations. However, causal evidence on the health effects of retirement based on a nationwide sample in China remains scarce. Considering hypertension is a major health risk among Chinese elderly, and blood pressure serves as a comprehensive health indicator with high predictive and preventive value for diseases, our study, based on a quasi-experimental design, provides robust causal associations between retirement and blood pressure in the Chinese population. We also offer health advice for several key subgroups, such as women, men, and medication users, benefiting their post-retirement health and welfare. Currently, optimizing retirement policies and delaying retirement are measures adopted worldwide to address the challenges of population aging, labor shortages, and financial pressures. However, the decision to delay retirement and how to optimize retirement policies should be supported by empirical evidence on the association between retirement and health. Based on our study's findings, we propose the following policy recommendations:

First, although we found retirement's impact on the overall population's blood pressure to be insignificant, significant effects were observed in specific subgroups. Moreover, the magnitude of effects varied significantly within different subgroups, sometimes even in opposing directions. Therefore, compared to a one-size-fits-all approach to delaying retirement, increasing the flexibility of retirement policies seems more rational. Implementing different extents of retirement age adjustments for diverse populations (e.g., men, women) may be more beneficial for public health. Second, policymakers and public health practitioners should pay extra attention to populations vulnerable to the adverse effects of retirement (such as those on antihypertensive medication and other chronic disease patients). Regular follow-ups on their health status could ensure adherence to treatment and maintain health post-retirement. Third, considering the low awareness rate among Chinese adult hypertension patients about their condition [49], community-based blood pressure screenings in retirement populations are necessary. This would contribute to reducing overall cardiovascular mortality [50] and improving the health status of the elderly.

### Limitation

This research has the following limitations. First, as a local regression approach, despite the strong internal validity of RDD, its external validity could be limited because the estimated causal impact of retirement was based on the participants close to the statutory retirement age. Thus, our results cannot be generalized to individuals who are far from the retirement age threshold. Second, changes in economic status could be a mechanism between retirement and health effects, but the CHS did not include the economic level of the participants such as housing conditions and income status. Therefore, economic-related covariates should be controlled in future studies on retirement’s health impact.

## Conclusions

Retirement is associated with an increase in blood pressure level. There is a causal relationship between the increase in blood pressure levels of men and retirement. Policy-makers should pay extra attention to the health status of men and less educated people when adjusting retirement policies in the future.

### Supplementary Information

**Supplementary Material 1.** 

## Data Availability

The data that support the findings of this study are available on reasonable request from the corresponding author.
