# Supplementary material for: The impact of retirement on blood pressure: evidence from a nationwide survey in China
Source: BMC Public Health. 2024 Jun 11;24:1565. doi: 10.1186/s12889-024-18422-z (PMC11165730; doi:10.1186/s12889-024-18422-z)
Supplement: Supplementary file 1 — Supplementary Material 1. [file 12889_2024_18422_MOESM1_ESM.doc]

# Supplementary materials

Table S1. The results of the first-stage regression of the FRDD.

| **Groups** | Coefficient | 95% CI | P value |
| --- | --- | --- | --- |
| **Full population** | 0.217 | (0.173,0.261) | 0.000 |
| **Male** | 0.226 | (0.161,0.291) | 0.000 |
| **Female** | 0.213 | (0.159,0.267) | 0.000 |
| **Low-educated** | 0.251 | (0.182,0.320) | 0.000 |
| **High-educated** | 0.188 | (0.132,0.244) | 0.000 |

Table S2. The results of the McCrary test for data manipulation.

|  | **Full population** | **Male** | **Female** | **Low-educated** | **High-educated** |
| --- | --- | --- | --- | --- | --- |
| **P value** | 0.358 | 0.138 | 0.901 | 0.269 | 0.857 |

Table S3. The continuity test of covariates was performed by using them as the outcome variable for the FRDD.

| **Covariate** | **Full participants** | | **Male** | | **Female** | | **Low-educated** | | **High-educated** | |
| --- | --- | --- | --- | --- | --- | --- | --- | --- | --- | --- |
|  | Coefficient (95% CI) | P value | Coefficient (95% CI) | P value | Coefficient (95% CI) | P value | Coefficient (95% CI) | P value | Coefficient (95% CI) | P value |
| **Married** | -0.005  (-0.089,0.079) | 0.903 | -0.019  (-0.126,0.088) | 0.731 | 0.008  (-0.117,0.134) | 0.895 | 0.017  (-0.095,0.130) | 0.762 | -0.031  (-0.159,0.096) | 0.629 |
| **Better educated** | -0.042  (-0.254,0.170) | 0.699 | -0.072  (-0.371,0.227) | 0.639 | -0.032  (-0.323,0.258) | 0.827 | - | - | - | - |
| **Smoke** | -0.055  (-0.239,0.130) | 0.562 | -0.133  (-0.438,0.172) | 0.394 | 0.046  (-0.028,0.120) | 0.220 | -0.137  (-0.409,0.135) | 0.324 | 0.016  (-0.238,0.270) | 0.903 |
| **Drink** | 0.073  (-0.093,0.239) | 0.388 | 0.170  (-0.123,0.463) | 0.255 | 0.000  (-0.128,0.128) | 0.997 | -0.042  (-0.286,0.203) | 0.738 | 0.174  (-0.060,0.408) | 0.146 |
| **Taking antihypertensive drugs** | -0.116  (-0.291,0.059) | 0.193 | 0.006  (-0.267,0.278) | 0.968 | -0.204  (-0.422,0.014) | 0.066 | 0.077  (-0.155,0.309) | 0.516 | -0.294  (-0.699,0.111) | 0.155 |

Table S4. The effect of retirement on blood pressure among different subgroups.

| **Group** | **Effect of retirement on SBP** | | **Effect of retirement on DBP** | | **Effect of retirement on pulse pressure** | |
| --- | --- | --- | --- | --- | --- | --- |
|  | Coefficient  (95% CI) | P value | Coefficient  (95% CI) | P value | Coefficient  (95% CI) | P value |
| **Male** | 14.980  (3.456,26.504) | 0.011 | 4.334  (-2.022,10.691) | 0.181 | 10.646  (2.290,19.002) | 0.013 |
| **Female** | -4.263  (-15.128,6.603) | 0.442 | -2.896  (-9.358,3.566) | 0.380 | -1.366  (-8.686,5.953) | 0.714 |
| **Low-educated** | 9.601  (-1.671,20.873) | 0.095 | 2.577  (-3.684,8.838) | 0.420 | 7.024  (-0.804,14.852) | 0.079 |
| **High-educated** | -0.359  (-11.631,10.914) | 0.950 | -1.799  (-8.755,5.157) | 0.612 | 1.440  (-6.447,9.328) | 0.720 |
| **Antihypertensive medication users** | 10.118  (-1.051,21.287) | 0.076 | 3.583  (-3.089,10.255) | 0.293 | 6.003  (-1.921,13.927) | 0.138 |
| **Non-antihypertensive medication users** | 1.772  (-6.495,10.040) | 0.674 | 0.340  (-4.555,5.235) | 0.892 | 1.431  (-4.353,7.215) | 0.628 |

Table S5. Quadratic terms for age and bias-corrected estimates with robust standard errors of the FRDD being used.

| **Groups** | **Model adjustment** | **Effect of retirement on SBP** | | **Effect of retirement on DBP** | | **Effect of retirement on pulse pressure** | |
| --- | --- | --- | --- | --- | --- | --- | --- |
|  |  | Coefficient  (95% CI) | P value | Coefficient  (95% CI) | P value | Coefficient  (95% CI) | P value |
| Full population | Quadratic terms | 7.636  (-5.192,20.464) | 0.243 | 1.434  (-6.028,8.897) | 0.706 | 6.201  (-2.872,15.275) | 0.180 |
|  | Bias-corrected estimates with robust standard errors | 7.355  (-4.080,18.790) | 0.207 | 1.338  (-5.378,8.055) | 0.696 | 6.017  (-2.044,14.077) | 0.143 |
| Male | Quadratic terms | 26.681  (-0.382,53.744) | 0.053 | 8.698  (-5.068,22.464) | 0.216 | 17.983  (-1.310,37.276) | 0.068 |
|  | Bias-corrected estimates with robust standard errors | 23.299  (6.630,39.968) | 0.006 | 7.437  (-1.730,16.603) | 0.112 | 15.862  (3.614,28.110) | 0.011 |
| Female | Quadratic terms | -7.508  (-23.798,8.781) | 0.366 | -4.981  (-14.749,4.788) | 0.318 | -2.527  (-13.501,8.447) | 0.652 |
|  | Bias-corrected estimates with robust standard errors | -7.444  (-23.307,8.418) | 0.358 | -4.940  (-14.401,4.521) | 0.306 | -2.504  (-13.263,8.254) | 0.648 |
| Low-educated | Quadratic terms | 8.077  (-11.631,27.786) | 0.422 | 4.495  (-6.340,15.329) | 0.416 | 3.583  (-10.058,17.223) | 0.607 |
|  | Bias-corrected estimates with robust standard errors | 8.334  (-8.192,24.860) | 0.323 | 4.171  (-4.717,13.060) | 0.358 | 4.163  (-7.469,15.795) | 0.483 |
| High-educated | Quadratic terms | 5.765  (-11.708,23.238) | 0.518 | -1.901  (-12.854,9.052) | 0.734 | 7.666  (-5.133,20.465) | 0.240 |
|  | Bias-corrected estimates with robust standard errors | 5.369  (-10.872,21.611) | 0.517 | -1.894  (-12.133,8.345) | 0.717 | 7.264  (-4.235,18.762) | 0.216 |


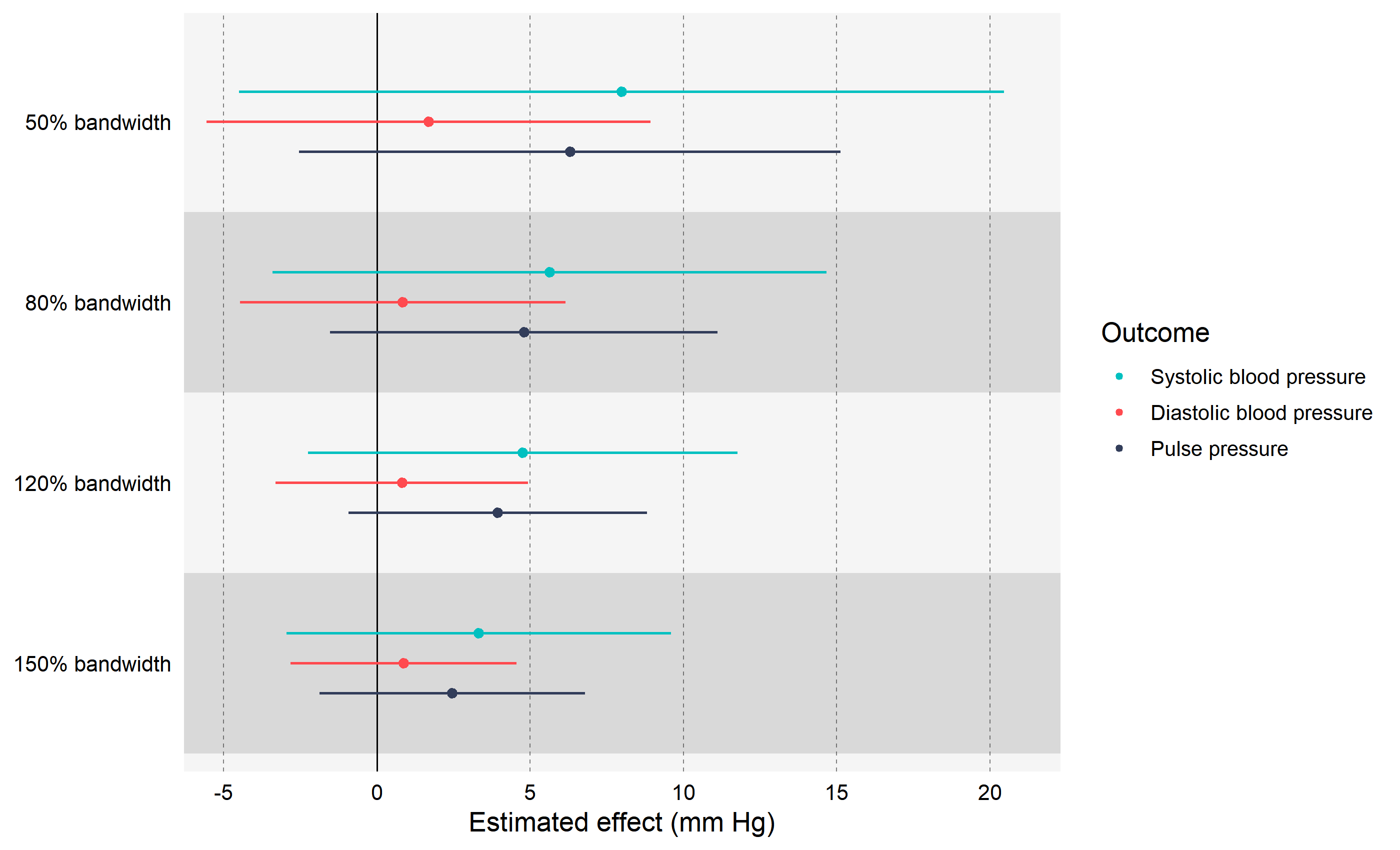


Figure S1. Using different bandwidths to estimate the effect of retirement on blood pressure.
